# Prediction of Impact Resistance of Nano-SiO_2_ and Hybrid Fiber Modified Geopolymer Gel Concrete in Marine Wet–Thermal and Chloride Salt Environment

**DOI:** 10.3390/gels12030215

**Published:** 2026-03-05

**Authors:** Canhua Lai, Peng Zhang, Xiaobing Dai, Yuanxun Zheng

**Affiliations:** 1 School of Water Conservancy and Transportation, Zhengzhou University, Zhengzhou 450001, China; 13395998071@163.com (C.L.); daixb@zzu.edu.cn (X.D.); yxzheng@zzu.edu.cn (Y.Z.); 2 State Key Laboratory of Tunnel Boring Machine and Intelligent Operation, Zhengzhou 450001, China

**Keywords:** geopolymer gel concrete (GPC), wet–thermal and chloride salt environment, RBF-BP composite neural network, impact resistance, prediction

## Abstract

The oceanic wet–thermal and chloride salt environment creates extremely harsh service conditions for marine infrastructures. As a green construction material, geopolymer concrete has a promising application prospect in marine engineering due to its excellent durability. The impact resistance of geopolymer concrete subjected to wet–thermal and chloride salt environment is of great significance for the durability and quality of marine engineering structures. This study uses nano-SiO_2_ (NS) and hybrid fibers (HF) to enhance the impact resistance of geopolymer gel concrete (GPC). Radial basis function (RBF) and back-propagation (BP) composite neural networks are used to predict the impact resistance of NS and HF-reinforced geopolymer gel concrete (NSHFGPC). The impact resistance of NSHFGPC specimens is characterized by two indicators: the cumulative number of repeated impact blows required to initiate the first visible crack (*N*_1_) and the cumulative number of impact blows corresponding to ultimate failure (*N*_2_). To evaluate the durability of NSHFGPC under oceanic conditions, specimens were exposed to a simulated marine environment within a simulation test chamber for 60 days prior to impact testing. The 60-day duration was selected to achieve a sufficient level of chloride penetration and matrix aging. Based on the resulting experimental database, an RBF-BP neural network was constructed to predict the material’s impact resistance. In this study, grid search and K-fold cross-validation were employed to select the optimal hyperparameters. Compared to standalone RBF and BP models, the RBF-BP network demonstrated superior performance, achieving *R*^2^ values of 0.900 and 0.922. These results represent improvements of 20.18% and 11.18% over the standalone RBF model, respectively. Consequently, the RBF-BP algorithm serves as an experimental tool for predicting NSHFGPC impact resistance and guiding future mix design optimization.

## 1. Introduction

Concrete is a composite material formed by mixing cementitious materials, aggregates, and water in appropriate proportions, followed by a hardening process. Concrete has become the most important building material in civil and hydraulic engineering due to its excellent mechanical properties, durability, and low cost [1,2]. Over the past few decades, the increasing demand for infrastructure and construction has doubled the global output of concrete, reaching approximately 26 gigatons by 2020 [3]. Taking the United States as an example, over 36,000 bridges are located within 15 miles of the coastline, and 32% of these reinforced concrete structures are suffering from severe degradation due to coupled marine wet–thermal and chloride salt environments [4]. Such statistics underscore the massive market demand for high-performance concrete specifically engineered for oceanic conditions. Cement functions as the primary binder within the concrete matrix and thereby integrates the constituent parts into a single entity [5]. A downside is that cement production releases substantial amounts of CO_2_ from fuel combustion and limestone decomposition, accounting for 5–8% of global human-caused emissions annually [6]. According to the World Business Council for Sustainable Development (WBCSD) Cement Sustainability Initiative (CSI) database, the production of one ton of clinker (a nodular material produced by fusing limestone and clay at high temperatures, serving as the core precursor for cement) generates approximately 840 kg of CO_2_, comprising 500 kg from limestone calcination and 300 kg from fuel combustion [7]. This has seriously polluted the environment, emitted a large amount of greenhouse gases, and exacerbated global warming [8]. Global warming has caused tremendous losses to human society and nature.

To alleviate environmental pollution problems caused by cement production, new studies are now seeking novel cement-free binding materials, such as geopolymers, that can completely substitute Ordinary Portland Cement (OPC) in concrete production. Qian et al. [9] gradually replaced cement with recycled construction waste-based cementitious materials and successfully developed an effective method for green ultra-high-performance concrete. This new type of cementitious material possesses both the advantages of environmental friendliness and high performance. Sadique et al. [10] conducted research on a new type of ternary blend composite cementitious material and found that this new material has good stability, high calcium content, and a low calcium-silicon ratio. Xu et al. [11] prepared an autoclaved aerated concrete using various concentrations of ammonia-soda residue (ASR) within a ground granulated blast furnace slag-steel slag-desulfurization gypsum (GGBS-SS-FGD) system. High production costs are the primary barrier preventing the three materials mentioned in this paragraph from being widely adopted.

Among all cement alternatives, geopolymer gel stands out as a promising binding material due to its high early strength, durability, low permeability, environmental friendliness, and cost-effectiveness [12,13,14]. Relative study results have confirmed the superior sulfate resistance of geopolymer gel concrete compared with ordinary Portland cement concrete [15,16]. Hussin et al. [17] discovered that the blended ash geopolymer gel concrete had better performance than ordinary Portland cement at high temperatures. When using geopolymer gel as the cementitious material in concrete production, the greenhouse gas emissions are reduced by approximately half compared to those of concrete with ordinary cement as the cementitious material [18]. However, geopolymer gel concrete still has some drawbacks, such as low flexural and tensile strengths, as well as relatively low density and a weak interface. To overcome these limitations, numerous strategies have been proposed for improving geopolymer gel concrete performance.

On the one hand, fibers are added to enhance the bending resistance and tensile resistance, such as polyvinyl alcohol (PVA) fiber [19,20], polypropylene fiber [21], basalt fibers [22], and steel fibers [23,24,25]. Deng et al. [26] observed that when PVA fibers are added to concrete mixes composed of slag and fly ash, the composite exhibits marked improvements in both flexural and splitting tensile strengths. Rashad et al. [27] found that adding steel fibers to the polymer matrix has a positive impact on its flexural strength, splitting tensile strength, ductility, fracture modulus, and elastic modulus. Hybrid fibers were also incorporated into the polymer concrete [28]. Gao et al. [29] studied the bonding properties of geopolymer gel concrete with different concentrations of steel fibers and PVA fibers. Therefore, steel fibers and PVA fibers are suitable as reinforcing materials for geopolymer gel concrete.

On the other hand, the addition of nanomaterials can enhance the aluminosilicate network of the geopolymer gel matrix, thereby refining the pore structure and densifying the matrix by filling interstitial voids [30,31,32]. Xavier et al. [33] conducted research on the impact of nano-alumina and graphite on geopolymer gel concrete and found that the strength of the geopolymer gel concrete was enhanced. Dong et al. [34] enhanced the strength of the geopolymer gel by using nano-SiO_2_ (NS). Ahmed [35] et al. found that when the NS content was 3%, it could effectively solve the problem of slow strength development and improve the mechanical and durability properties. Therefore, the addition of NS and mixed fibers can significantly enhance the performance of geopolymer concrete.

Every country attaches great importance to its marine interests. The exploitation of marine resources and the expansion of human activities from land to sea require a large amount of infrastructure, such as military ports, offshore oil platforms, cross-sea bridges, offshore airports, and lighthouses, and all these infrastructures require huge amounts of concrete [36]. Marine concrete infrastructure is subjected to severe deterioration due to the ingress of salt ions like chloride and sulfate [37,38]. The dry–wet cycle [39,40] and the high temperatures in tropical and subtropical regions have accelerated this process. Therefore, it is of great significance to find a type of concrete that can perform well under the combined influence of oceanic wet–thermal and chloride salt environment. Geopolymer gel concrete performs better than ordinary cement concrete in the face of a wet, hot, and saline coupled marine environments; it can better resist the erosion caused by ions such as chloride in seawater [41,42,43]. Therefore, geopolymer gel concrete has significant advantages for service in wet–thermal and chloride salt environments.

Machine learning has developed rapidly in recent years, and the same is true in the field of building materials. Machine learning technology based on existing experimental data for predicting the performance of building materials can significantly reduce the human, material, and financial resources required for performance experiments of building materials. It can also provide guidance for the design of concrete mix ratios and engineering research [44,45]. Yan et al. [46] predict the bonding strength of concrete based on an artificial neural network optimized by genetic algorithms (GA). Abd et al. [47] uses support vector machine (SVM) to predict the compressive strength of lightweight foam concrete. The scholars also used K-Nearest Neighbors (KNN) [48], Radial Basis Function (RBF) [49], and Back Propagation Neural Network (BP) [50,51,52,53] respectively to predict the performance of concrete.

Despite the rapid development of machine learning in construction materials, a significant research gap exists. Current studies primarily focus on predicting the basic mechanical properties of geopolymer concrete under terrestrial environments. However, there is a scarcity of research on the impact resistance of NS and hybrid fiber (HF) reinforced geopolymer gel concrete (NSHFGPC) specifically subjected to the coupled effects of oceanic wet–thermal and chloride salt environments. Furthermore, a predictive method capable of handling the complex, small-sample, and nonlinear relationships inherent in oceanic coupled conditions remains underdeveloped. Therefore, the RBF-BP neural network is used to predict the impact resistance of NSHFGPC subjected to oceanic wet–thermal and chloride salt environments. It will also lay a foundation and provide a reference for the mix design and engineering research of geopolymer gel concrete in the future.

## 2. Results and Discussion

In order to conduct a more comprehensive analysis of the prediction results, this study compares the predicted values of the RBF-BP, RBF, and BP with the actual values. In this study, the output data consists of 13 sets of samples. To comprehensively evaluate the fitting and prediction performance of the three models, the figure shows the comparison between the predicted values and the actual values of the three models rather than separately presenting the validation set. Compared to presenting the validation set alone, this approach can more intuitively demonstrate the model’s predictive ability for the entire dataset in cases of small sample sizes and avoid selection bias. When the sample size is small, presenting the entire sample can better reflect the fitting trend of the model. By presenting the full dataset, the model’s predictive capacity can be comprehensively demonstrated, and the objectivity and reliability of the results can be guaranteed.

**Figure 1:**
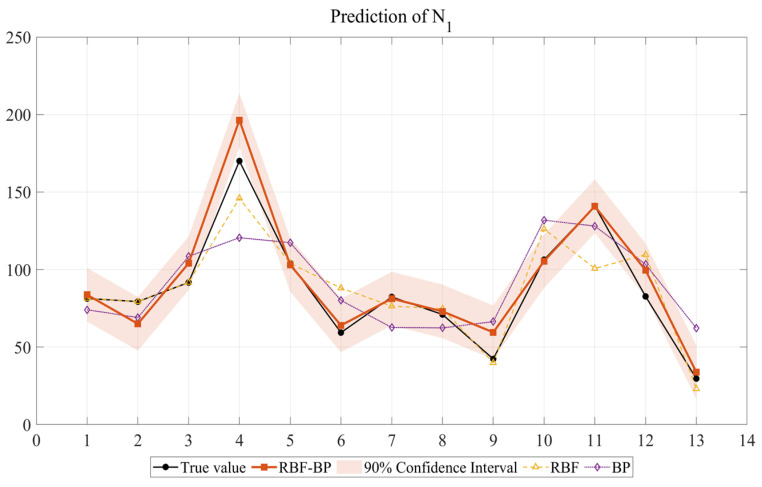
Prediction of *N*_1_.

**Figure 2:**
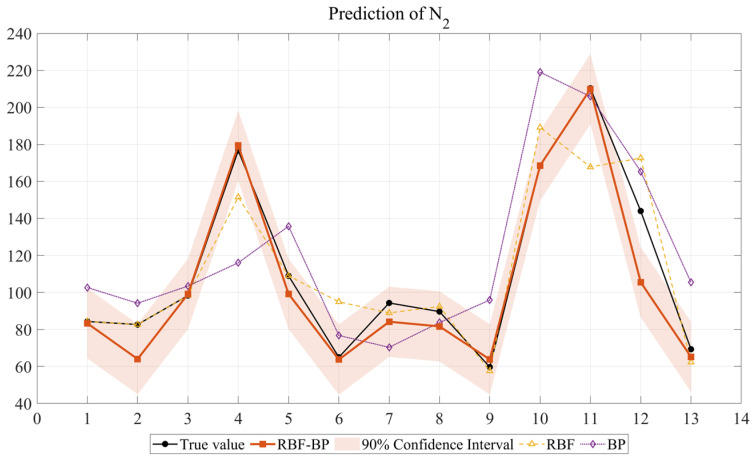
Prediction of *N*_2_.

After model construction and prediction, the prediction results of the forecasting outcomes of the three neural networks for the impact resistance prediction indicators of NSHFGPC are shown in Figure 1 and Figure 2, respectively. Each of the 13 concrete mix groups is assigned a ‘mix number’ as a unique identifier. This facilitates a clearer comparison of the neural network prediction results across all NSHFGPC specimens. The correspondence between the specimens and the mix number is shown in Table 1.

To further evaluate the statistical reliability and uncertainty of the proposed model under limited sample conditions, a 90% confidence interval analysis was performed for the RBF-BP predictions. As illustrated in Figure 1 and Figure 2, the experimental data points for both the cumulative number of repeated impact blows required to initiate the first visible crack of NSHFGPC (*N_1_*) and the cumulative number of impact blows corresponding to ultimate failure of NSHFGPC (*N*_2_) fall consistently within the shaded confidence bands. The narrow width of the confidence intervals demonstrates high prediction stability. The hybrid network maintains reliability despite the small sample size. The mathematical framework effectively quantifies potential variations. Experimental data points fall consistently within the shaded confidence bands. This uncertainty analysis provides evidence that the RBF-BP model is reliable for predicting the impact resistance of the investigated geopolymer mortar.

In Groups 1 to 5, the NS contents were 0%, 0.5%, 1.0%, 1.5%, and 2.0%, respectively. No PVA fibers or steel fibers were added. Figure 1 and Figure 2 show that the *N*_1_ and *N*_2_ of NSHFGPC for Groups 1–5 exhibit an initial increase followed by a decline as the NS content increases. The two characteristics of NS are responsible for the above phenomenon. First, the good filling effect and reactivity of NS can fill the internal voids of the concrete [54], optimizing the interfacial transition zone performance between NSHFGPC cementitious material and coarse aggregate. Thereby, NS can enhance the mechanical properties of the NSHFGPC. Second, the NS will agglomerate when the content of NS is too high, thereby reducing the mechanical properties of the concrete [55]. The effect of these two properties on the NSHFGPC is strong or weak, and it is manifested as an increase or decrease in the impact resistance of the NSHFGPC on a macroscopic level. When the content of NS ranges from 0% to 1.5%, the first property dominates. The first property dominates the position, which persists until the content of NS reaches 1.5%. At this point, both *N*_1_ and *N*_2_ of the NSHFGPC reach their highest. Subsequently, the second property dominates, so the impact resistance of the NSHFGPC decreases.

**Figure 3:**
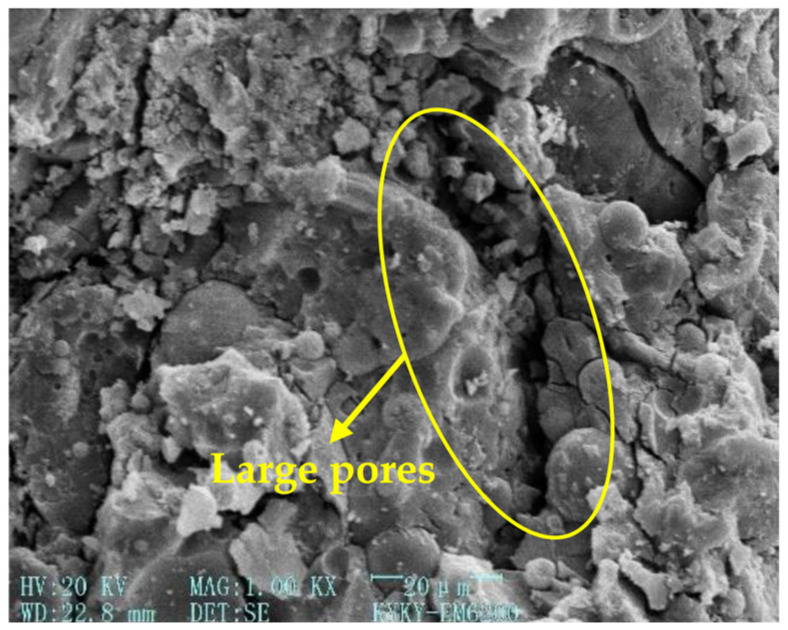
NS dosage: 0% (adapted from [56]).

**Figure 4:**
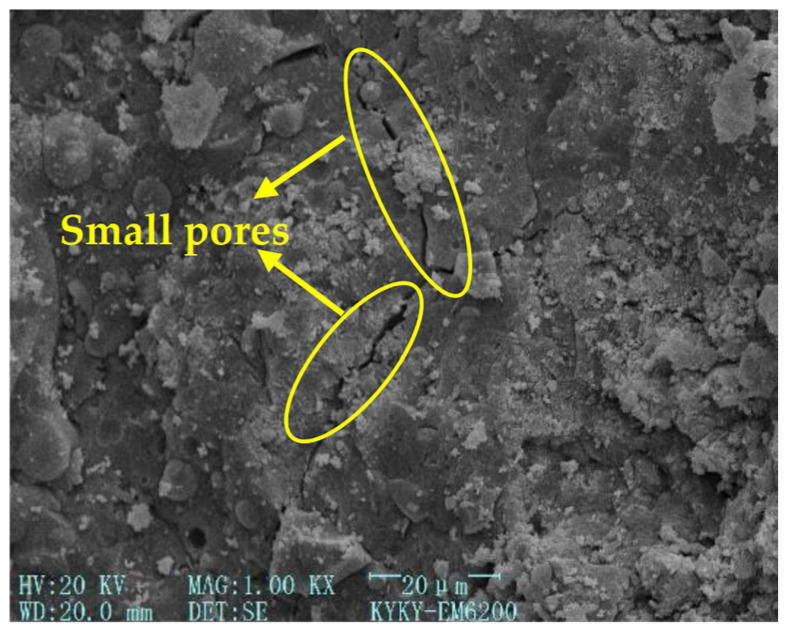
NS dosage: 0.5% (adapted from [56]).

**Figure 5:**
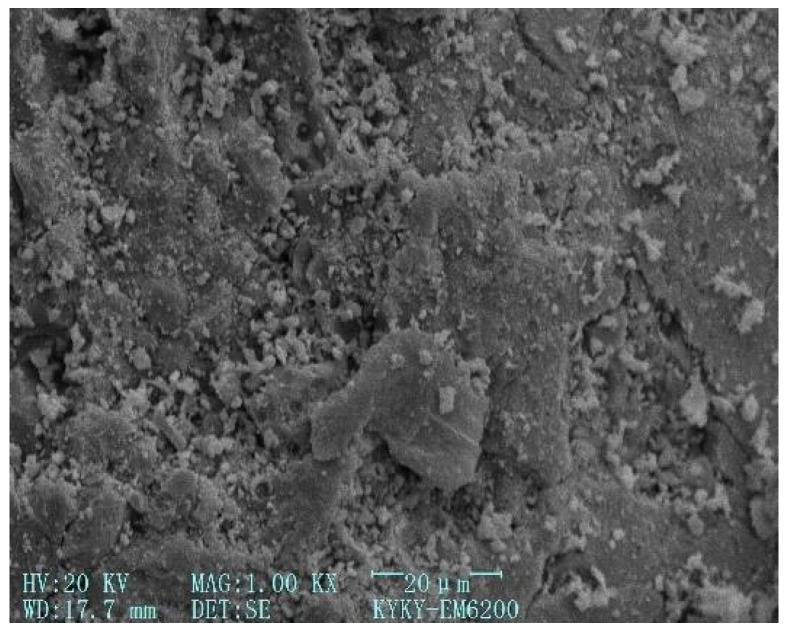
NS dosage: 1.5% (adapted from [56]).

**Figure 6:**
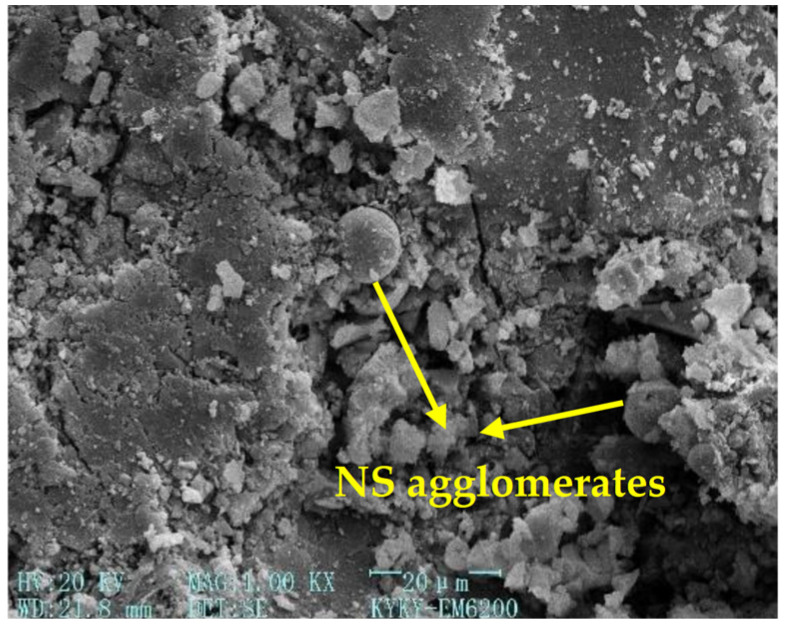
NS dosage: 2.0% (adapted from [56]).

Figure 3, Figure 4, Figure 5 and Figure 6 [56] illustrate the microstructural morphologies of the NSHFGPC specimens containing 0%, 0.5%, 1.5%, and 2.0% NS, respectively. The scanning electron microscope (SEM) analysis distinguishes the filling effect when the content of NS was low (0–1.5%) and the agglomeration effect when the content of NS was high (2.0%).

NS significantly promotes the hydration reaction of MK within the geopolymer matrix. At lower NS dosages (0.5–1.5%), the relatively abundant MK reacts with NS to generate a substantial amount of calcium aluminosilicate hydrate (C-A-S-H) gel, which effectively exerts a ‘filling effect’ by occupying the micro-pores within the NSHFGPC matrix. This leads to a refined and smoother microstructure, as observed in Figure 4 and Figure 5. Specifically, Figure 5 reveals the densest matrix with minimum porosity at a 1.5% NS dosage, representing the optimal threshold for mechanical enhancement. Conversely, when the NS content is excessive (2.0%), the high specific surface energy of the nanoparticles causes significant agglomeration. As shown in Figure 6, these clusters create localized structural defects and increase internal porosity, thereby degrading the impact resistance of the NSHFGPC specimens.

For groups 6–9, the content of NS is fixed at 1.5%, and the content of PVA fibers ranges from 0.2% to 0.8%. For groups 10–13, the content of NS is fixed at 1.5%, the content of steel fibers is fixed at 1%, and the content of PVA fibers ranges from 0.2% to 0.8%. In groups 6–9 and 10–13, as fiber content increased, *N*_1_ and *N*_2_ showed a trend of initial increase followed by a decrease. For groups 6–9, the increased fiber content corresponds to more PVA fibers. For groups 10–13, the increased fiber content corresponds to a higher total amount of PVA and steel fibers in the mixed fiber system. The trend of impact resistance was the same: as the fiber content increases, it initially rises and then decreases. This phenomenon is mainly due to two reasons. First, both PVA [57] fiber, steel fiber [58], and the mixed fibers can enhance the impact resistance of NSHFGPC when it is subjected to external impact by the adhesive effect of the fibers on the aggregate and the tensile strength of the fibers themselves. As a result, the impact resistance of the NSHFGPC increases. Second, the excess high fiber content will reduce the density and uniformity of the concrete, and cause the deterioration of the concrete [59] in an oceanic wet–thermal and chloride salt environment. As a result, the impact resistance of the NSHFGPC decreases. As the fiber content increased, the first effect dominated when the PVA content was 0.2–0.4% in the 6–9 groups. *N*_1_ and *N*_2_ reached the maximum when the PVA content was 0.4%. Then the second effect dominated, showing a decrease in impact resistance when the PVA content was 0.4–0.8%. In the 10–13 groups, when the content of the mixed fibers (the sum of the PVA fiber content and the steel fiber content) was 1.4%, the impact resistance reached the optimal level. The enhancement in the impact resistance of NSHFGPC through the integration of mixed fibers is attributed to the synergistic effect between PVA fibers and steel fibers. At the crack initiation stage, the highly flexible PVA fibers effectively inhibit the nucleation and early evolution of micro-cracks by distributing the stress at the crack tip to the surrounding matrix. As the impact load persists, micro-cracks converge into macroscopic cracks, where the high-strength steel fibers become dominant. By leveraging superior anchoring force, the steel fibers exert a “bridging effect” that significantly restricts further crack propagation, thereby substantially delaying the ultimate failure of the NSHFGPC structure.

The deterioration of NSHFGPC in the oceanic wet–thermal and chloride salt environment stems from two primary aspects. On the one hand, compared to terrestrial conditions, the marine environment is characterized by high temperature, humidity, and high chloride ion concentration. These environmental factors aggressively attack the internal structure of NSHFGPC through original surface micro-cracks, leading to the deterioration of NSHFGPC and a decrease in the strength of NSHFGPC. On the other hand, the degradation is linked to the constituents, specifically NS and HF. With prolonged exposure, the filling effect of NS on NSHFGPC diminishes, and the internal pores of NSHFGPC increase. Furthermore, the inclusion of HF introduces interfacial defects within NSHFGPC. These interfacial defects provide additional pathways for chloride ions and water molecules to infiltrate the NSHFGPC matrix. Consequently, the ingress of chloride ions and water molecules accelerates the deterioration of NSHFGPC. Additionally, the corrosion of steel fibers in the oceanic wet–thermal and chloride salt environment acts as a significant factor contributing to the performance decline of NSHFGPC.

The comparison of the prediction performance of three neural networks is shown in Table 2. This study obtained the performance evaluation indicators by comparing the predicted values with the actual values. These indicators consist of four parameters: *R*^2^, *RMSE*, *MAPE*, and *MAE*, which correspond to the coefficient of determination, root mean squared error, mean absolute percentage error, and mean absolute error, respectively.

*R* ^2^ *:*

(1) $$\begin{array}{c} R^{2}=1-\frac{\sum_{i=1}^{n}\;(y_{i}-{\hat{y}}_{i}{)}^{2}}{\sum_{i=1}^{n}\;(y_{i}-\bar{y}{)}^{2}} \end{array}$$

*RMSE:*

(2) $$\begin{array}{c} RMSE=\sqrt{\frac{1}{n}\sum_{i=1}^{n}\;(y_{i}-{\hat{y}}_{i}{)}^{2}} \end{array}$$

*MAPE:*

(3) $$\begin{array}{c} MAPE=\frac{100\%}{n}\sum_{i=1}^{n}\;\left|\frac{y_{i}-{\hat{y}}_{i}}{y_{i}}\right| \end{array}$$

*MAE:*

(4) $$\begin{array}{c} MAE=\frac{1}{n}\sum_{i=1}^{n}\;|y_{i}-{\hat{y}}_{i}| \end{array}$$

As shown in Figure 1 and Figure 2 and Table 2, the RBF-BP neural network achieves the optimal prediction performance, followed by the RBF neural network, with the BP neural network showing the poorest results.

The RBF-BP network performed best, with the two output variables reaching 0.9003 and 0.9222, respectively. The *R*^2^ values of the two output variables of the RBF-BP neural network are 20.18% and 11.18% higher than those of the RBF neural network, indicating that the RBF-BP neural network achieves a better and more accurate fit to the data. This performance can be attributed to the intrinsic characteristics of the RBF-BP network. The composite architecture first employs the RBF layer for nonlinear feature mapping, capturing local patterns effectively. Subsequently, the composite architecture utilizes the BP layer for global optimization, improving the overall fitting capability. Owing to this hybrid mechanism, the RBF-BP network demonstrates better performance compared to standalone RBF and BP neural networks. The RBF-BP model achieves an incremental enhancement in accuracy compared to the standalone RBF. However, the experimental data of this study only consists of 13 sets, which is a relatively small sample size for the training of neural networks. The neural network trained based on this data has limitations. The small sample size is partly due to the lengthy cycle of the concrete experiments and the limitation on the number of samples that can be produced at one time by the experimental setup used to simulate the oceanic wet–thermal and chloride salt environment.

The RBF neural network also performed well, but it was inferior to the RBF-BP composite neural network. It has an *R*^2^ value of 0.7491 for the number of *N*_1_ and 0.8294 for *N*_2_. This might be related to the nature of the RBF network: its local approximation property enables relatively accurate modeling of detailed nonlinear relationships. However, the lack of global optimization capability limits its performance when dealing with complex patterns or larger datasets, thereby reducing its overall predictive robustness.

The BP neural network performed the worst in this study. BP neural network has a prediction accuracy far lower than that of the RBF and RBF-BP. The performance disparity between the standalone BP and RBF models stems from the interaction between the limited sample size (13 sets) and the high nonlinearity of NSHFGPC datasets. In small-sample scenarios, BP networks are prone to initialization sensitivity and suboptimal convergence. In contrast, RBF networks utilize radial basis functions to construct an intermediate feature space that effectively captures local response patterns, offering superior stability. This gap provides the rationale for the RBF–BP hybrid sequence, which leverages RBF for feature representation and BP for subsequent parametric learning.

It should be noted that the developed RBF-BP model is characterized as an exploratory predictive tool tailored to the specific experimental range of this study, providing a reliable framework for mix-design optimization under limited data conditions.

## 3. Conclusions

In this study, two parameters of the impact resistance of NSHFGPC under wet–thermal and chloride salt environment were predicted using three neural networks. These parameters are *N*_1_ and *N*_2_. It should be noted that the developed RBF-BP model is characterized as an exploratory predictive tool tailored to the specific experimental range of this study, providing a reliable framework for mix-design optimization under limited data conditions. The following are the main conclusions of this study.

(1) Among the prediction accuracy of the three neural networks, the RBF-BP network exhibited the optimal performance. The *R*^2^ of the two output variables reached 0.9003 and 0.9222, respectively, while the *MAPEs* were 10.6732% and 7.4373%, respectively. The fitting effect was acceptable. This may be owing to the RBF-BP, this hybrid mechanism. The RBF-BP network demonstrates improved performance compared to the standalone RBF and BP networks.
(2) The RBF neural network also demonstrates excellent prediction performance. The *R*^2^ of the two output variables reached 0.7491 and 0.8294, respectively. The fitting effect of BP is the worst. This might be because the BP network has limited learning ability on small sample nonlinear data and is prone to getting stuck in local minima or underfitting in cases of small sample size or strong nonlinearity. The RBF-BP combined structure provides a more comprehensive and flexible modeling framework.
(3) Comparative analysis under small-sample conditions (N = 13) confirms the superiority of the RBF-BP model over traditional MLR and SVR. While MLR failed due to rank deficiency and multicollinearity, and SVR suffered from underfitting, the RBF-BP architecture successfully captured complex nonlinear relationships. This proves RBF-BP is a reliable exploratory predictive tool for concrete performance optimization when experimental data is limited.
(4) This study suggests that the RBF-BP neural network can assist engineers in predicting concrete mix proportions under marine wet–thermal and chloride salt environments. The RBF-BP neural network is suitable for small sample predictions and can reduce the workload of engineers. Concrete experiments are extremely time-consuming, and the curing time for concrete test specimens can be measured in months. If special environments are also simulated, the time for simulating the environment can also be measured in months. The test cycle is as short as three months and as long as six months.
(5) It is recommended to prioritize a composite reinforcement system combining hybrid fibers with NS, rather than relying on single-fiber reinforcement. As evidenced by the NSP-0.4 group achieving the highest *N*_2_ and *N*, the synergistic effect of co-incorporating NS and hybrid fibers maximizes the energy absorption capacity of the matrix prior to failure, ensuring superior structural safety in extreme environments.

## 4. Materials and Methods

### 4.1. Experiment Program

In fabricating the NSHFGPC, a control variable approach was adopted. In the control variable method of this study, cement–sand ratio, water–binder ratio, and modulus of alkali activator are maintained constant. Following trial mixing and adjustments, the proportions of metakaolin (MK) and fly ash (FA) utilized in this experiment were set at a 6:4 ratio. Additionally, the alkali activator modulus employed was 1.3. The molarity of the sodium hydroxide (NaOH) solution was adjusted to approximately 16.7 mol/L. The mass ratio of sodium silicate to NaOH was fixed at 5.38. For NSHFGPC, the water-to-binder ratio was set at 0.52. The binder-to-aggregate and sand ratios were fixed at 3.0 and 0.35, respectively.

The primary raw materials employed in this study include FA, MK, NS, PVA fibers, steel fiber microfilaments, sodium silicate, NaOH, river sand, graded crushed stone, and water. All materials meet the relevant standards and are suitable for the experimental procedures. The specific conditions of each raw material are as follows.

To ensure the reproducibility of the study, the processing methods for the precursors were specified. The MK used was a commercial-grade product prepared by calcining high-purity raw kaolin within a temperature range typical for dehydroxylation (generally between 700 °C and 900 °C). This thermal treatment ensures the conversion of kaolin into a highly reactive amorphous state. The calcined MK was subsequently subjected to mechanical grinding to achieve a fine powder consistency suitable for geopolymerization. The FA was a low-calcium Class F type, sourced from Luoyang Power Plant and collected via standard electrostatic precipitation. The precursors were used in their as-received state to maintain mineralogical consistency throughout the experiments. The chemical compositions and physical properties of MK and FA are summarized in Table 3, Table 4 and Table 5. Detailed properties of NS are summarized in Table 6, an amorphous white powder supplied by Wanjing New Materials Co., Ltd. (Hangzhou, China). The PVA fibers used in this study were produced by Kuraray Co., Ltd. (Tokyo, Japan)., Japan, and the physical properties of PVA are displayed in Table 7. The steel fibers used in this experiment have smooth outer surfaces and lengths ranging from 12 to 14 mm, with diameters of approximately 0.2 mm. A standard industrial-grade sodium silicate solution was used. The industrial-grade sodium silicate solution appears as a viscous light-yellow liquid, with its solid content and density measured to be 40% and 1.41 g/cm^3^, respectively. NaOH was added to reduce the modulus of water glass (the ratio of SiO_2_ to Na_2_O) from 3.2 to 1.3, according to Sun et al. [60]. The NaOH is a pure white, granular solid with a purity of 99.0%. Coarse aggregate was graded crushed stone with particle sizes ranging from 5 to 20 mm, while fine aggregate consisted of medium river sand with a fineness modulus of 2.8.

Four mix series were formulated to examine the individual and combined effects of hybrid fiber (HF) and NS on the impact resistance of NSHFGPC. Among them, PVA fibers are added based on volume concentration, while NS is added based on mass ratio.

**Figure 7:**
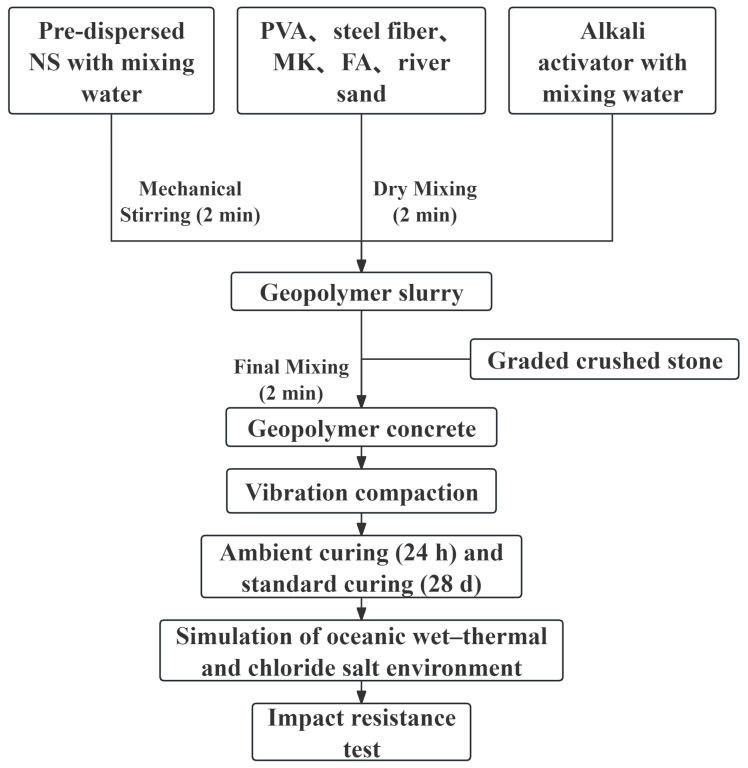
The preparation process of the NSHFGPC specimen.

The preparation of NSHFGPC specimens was conducted in a 50 L mechanical mixer. Prior to mixing, the alkali activator was prepared and cooled to room temperature to dissipate the heat generated by the exothermic dissolution of NaOH. Simultaneously, to mitigate the agglomeration of nanoparticles, the NS was pre-dispersed in the additional mixing water via high-speed mechanical stirring for 2 min. The mixing procedure commenced with the dry mixing of steel fibers, PVA fibers, precursors (MK and FA), and river sand for 2 min to ensure the effective separation and uniform distribution of the fibers. Subsequently, the cooled alkali activator and the NS suspension were introduced to the dry mixture and agitated for 1 min to form a cohesive geopolymer slurry. Finally, the coarse aggregates were added, followed by a final mixing period of 2 min to achieve a homogeneous fresh geopolymer gel concrete. Upon completion, the concrete was cast into molds, compacted using a vibrating table, and sealed with plastic film to prevent moisture loss. The NSHFGPC specimens were maintained at 20 ± 5 °C with a relative humidity above 95% during the initial 24 h of ambient curing. After this initial period, the NSHFGPC specimens were demolded and transferred to a standard curing room for 28 days. Subsequently, the NSHFGPC specimens were subjected to a simulated oceanic wet–thermal and chloride salt environment and impact resistance testing. In accordance with the standards GB/T 50081-2016 [61] and CECS 13-2009 [62], the dimensions of the NSHFGPC specimens were ultimately determined as 100 mm × 100 mm × 100 mm cubes. Figure 7 illustrates the preparation process of the NSHFGPC specimen and the subsequent experimental procedures.

The impact resistance of NSHFGPC specimens is characterized by two indicators: the cumulative number of repeated impact blows required to initiate the first visible crack (*N*_1_), and the cumulative number of impact blows corresponding to ultimate failure (*N*_2_). The two indices represent the number of impacts when the first crack appears and the number of impacts corresponding to specimen damage, respectively. Damage is defined as the formation of visible cracks and the subsequent complete fracture of the specimen under repeated impact loading. While compressive and tensile strengths are essential baseline properties for concrete materials, the durability of concrete structures in oceanic wet–thermal and chloride salt environments is often governed by repetitive dynamic impact damage rather than static load capacity. In splash and tidal zones, impact-induced microcracking accelerates chloride ingress and reinforcement corrosion, leading to premature deterioration. Therefore, this study focuses on impact resistance as the primary performance indicator to characterize the dominant damage mechanism under marine service conditions. *N*_1_ and *N*_2_ were calculated using the truncated average method (TAM). Impact toughness (*N*) of the geopolymer gel concrete is described by *N*_2_ minus *N*_1_, which can be represented by Equation (5).

(5) $$N=N_{2}-N_{1}$$

The two indicators for evaluating the impact resistance of NSHFGPC mentioned above exhibit the following trends: The higher *N*_1_ and *N*_2_, the better the impact resistance of NSHFGPC. The greater the difference between *N*_1_ and *N*_2_, the higher *N*, which indicates better impact resistance of NSHFGPC. Table 8 presents the mix proportions of NSHFGPC corresponding to the test set. The impact toughness of NSHFGPC can be quantified through both the *N* and the total energy absorption. In this study, since each blow from the drop weight delivers a constant amount of impact energy, the value of *N* is directly proportional to the total energy absorbed by the specimen during the crack propagation stage. Consequently, Equation (5) is employed to evaluate the impact toughness of NSHFGPC by calculating the difference between the *N*_1_ and the *N*_2_. This metric effectively represents the capacity of the material to dissipate energy after the onset of cracking. Table 9 gives *N*_1_, *N*_2_, and *N* in experiment.

The high variability in Table 9, particularly the low N_1_ and N_2_ for NP-0.8 and NSP-0.8, is attributed to localized fiber agglomeration. Inconsistent dispersion creates fiber clusters that act as structural voids within the NSHFGPC matrix. These defects induce stress concentration under impact loading, leading to premature crack initiation and the observed data heterogeneity.

### 4.2. Simulation of the Wet–Thermal and Chloride Salt Environment

**Figure 8:**
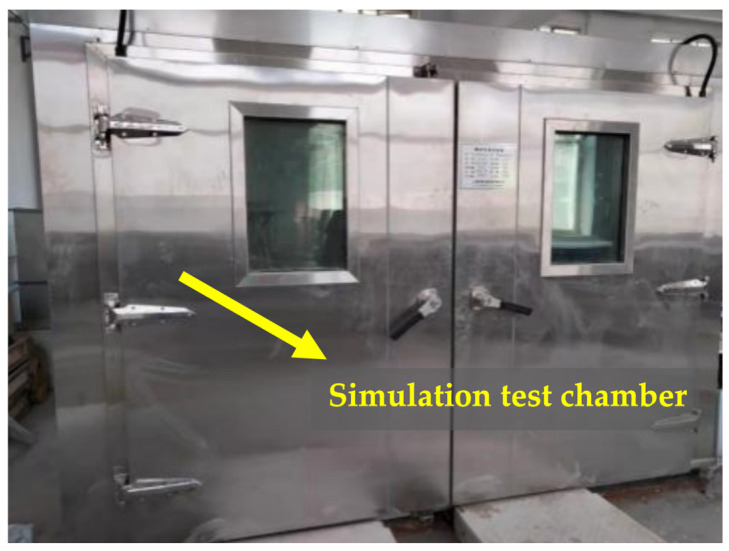
Appearance of the simulation test chamber.

**Figure 9:**
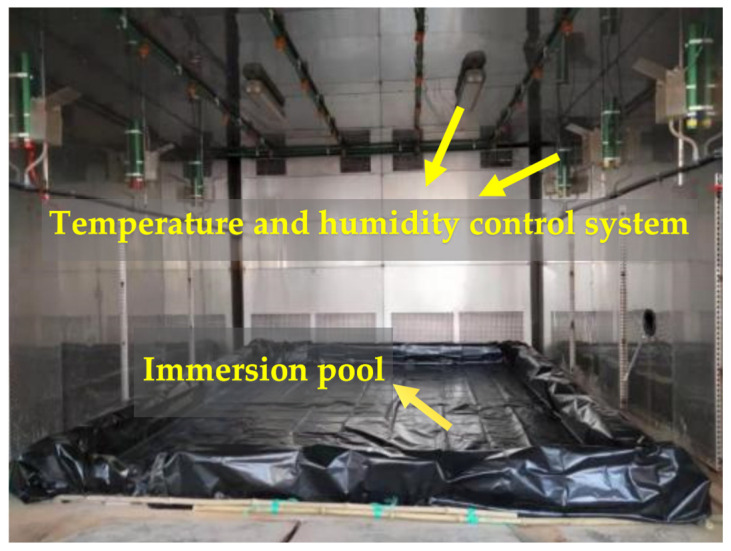
Internal structure of the simulation test chamber.

Taking into account the limitations of the laboratory conditions, the time constraints, and the need for experimental reproducibility, this study simulates the marine environment by placing the NSHFGPC specimens in a simulation test chamber. After a simulation in the marine environment, the NSHFGPC specimens were removed from the test chamber and subjected to an impact test. In order to test the impact resistance of concrete under the influence of the humid and salty marine environment, it is very important to select the test chamber simulated marine environmental parameters. This study referres to the seawater immersion environment in the southeastern coastal areas of China, and took the temperature, humidity, and chloride ion concentration as the factors for the test chamber simulation of concrete’s exposure to the marine environment. Figure 8 and Figure 9 illustrate the structure of the simulation test chamber.

To simulate the coupled environment, the relative humidity was maintained at 100%. The exposure temperature was set at 45 °C. A 5% chloride salt solution was utilized for the immersion process. The total duration of the environmental exposure lasted 60 days. The 60-day duration was selected to achieve a sufficient level of chloride penetration and matrix aging as per previous studies on accelerated marine testing. In this study, the methods of spraying and immersing in salt solution were employed to maintain a constant relative humidity and chloride salt concentration. To achieve the accelerated effect within the simulation test chamber, during the entire 60-day experiment period, it was ensured that each experimental specimen underwent 10 dry–wet cycles. One dry-wet cycle lasted for six days, during which the specimen was immersed in the chloride salt solution for 3 days and then air-dried for 3 days.

### 4.3. Model Establishment of RBF and BP Neural Network

To achieve the aim of predicting impact resistance and minimizing the error, a REF-BP composite neural network was applied in the prediction process. In order to comprehensively complete the construction of the prediction model, seven parameters serve as the input parameters for the model, namely the proportions of NS, PVA fibers, steel fibers, MK, FA, coarse aggregate, and fine aggregate. Two parameters that characterize the impact resistance of NSHFGPC were selected as the output parameters. The two parameters are *N*_1_ and *N*_2_. Therefore, both the BP and RBF neural networks are designed to include an input layer containing seven neurons and an output layer containing two neurons.

In this study, Matlab R2023a was used to construct the program. Some of the hyperparameters are determined through grid search, while the remaining parameters are determined based on experience. Key hyperparameters were obtained by grid search to eliminating the labor-intensive manual tuning process, while the remaining parameters were set empirically to avoid the excessive runtime associated with an exhaustive grid search. The components of the grid search parameters are shown in Table 10. All computational models were implemented in the MATLAB environment. The Deep Learning Toolbox was used to construct and train the RBF-BP hybrid model as well as the standalone neural networks, including RBF and BP models, through functions such as newrb, feedforwardnet, train, and mapminmax. The Statistics and Machine Learning Toolbox was employed to develop the benchmark models, including support vector regression (SVR) and multiple linear regression (MLR), using the functions fitrsvm and fitlm. Standard MATLAB built-in functions were utilized for data preprocessing, normalization, statistical analysis, and visualization.

#### 4.3.1. RBF Neural Network

Proposed by Lowe et al. [64] in 1988, the RBF network is a feedforward neural network consisting of an input layer, a hidden layer, and an output layer. Due to the universal approximation capability of the RBF network for continuous functions [65], the RBF network has been successfully adopted in diverse engineering domains [66,67]. The input layer consists of neurons that relay received signals to the hidden layer without any processing. The hidden layer utilizes a radial basis function as its activation (kernel) function to perform spatial mapping. The output layer uses a linear activation function, representing a linear combination of the hidden layer’s outputs. Thus, the transformation from the input layer to the hidden layer is nonlinear, while the transformation from the hidden layer to the output layer is linear [68].

The RBF component of the hybrid RBF-BP network is detailed below. Common radial basis functions include the Gaussian function and the inverse multiquadric function. In this study, the most commonly used Gaussian function is adopted.

The activation matrix of the RBF hidden layer, the linear mapping of the output layer, and the loss function (with regularization) are as shown in Equations (6)–(9).

(6) $$H=\left[\begin{array}{cccc} h_{1}(x_{1}) & h_{2}(x_{1}) & \ldots & h_{N_{h}}(x_{1})\\ h_{1}(x_{2}) & h_{2}(x_{2}) & \ldots & h_{N_{h}}(x_{2})\\ \vdots & \vdots & \ddots & \vdots \\ h_{1}(x_{n}) & h_{2}(x_{n}) & \ldots & h_{N_{h}}(x_{n}) \end{array}\right]$$

(7) $$h_{i}(x_{j})=\varphi_{i}(\left\|x_{j}-c_{i}\right\|)=\exp(-\frac{{\left\|x_{j}-c_{i}\right\|}^{2}}{2{\sigma_{i}}^{2}}),\;i=1,\;2,\;\ldots ,\;N_{h}$$

(8) $$\hat{Y}=HW+1_{n}b^{⊤}$$

(9) $$L=\frac{1}{n}∥\hat{Y}-Y{∥}_{F}^{2}+\lambda ∥W{∥}_{F}^{2}$$

where $x_{j}\&\in R^{1\times d}$ is the *j* th input sample, n is the total number of samples, d is the input feature dimension, $N_{h}$ is the number of RBF hidden layer neurons, $c_{i}\&\in R^{1\times d}$ is the center of the *i* th RBF neuron, $\sigma_{i}$ is the width of the *i*th RBF neuron, $H\in R^{n\times N_{h}}$ is the hidden layer output matrix, $W\&\in R^{N_{h}\times m}$ is the output weight matrix, $b\&\in R^{m\times 1}$ is the output bias, $1_{n}\&\in R^{n\times 1}$ is the all 1 column vector, $\hat{Y}\&\in R^{n\times m}$ is the predicted output, m is the output dimension. $Y\in R^{n\times m}$ is actual output, $∥\cdot {∥}_{F}$ is Frobenius norm, and λ is regularization coefficient, used to prevent overfitting.

**Figure 10:**
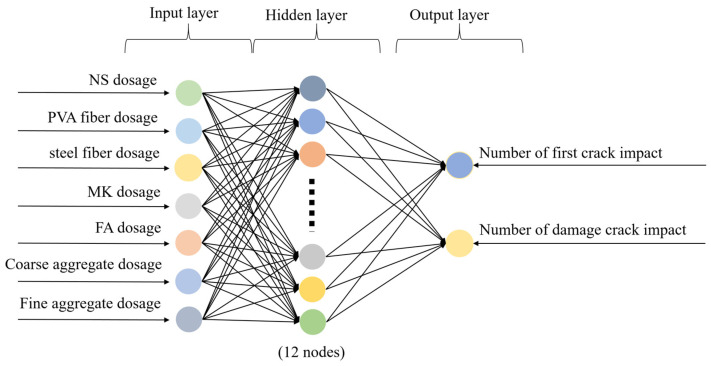
Structure of REF neural network.

When selecting the hyperparameters, the number of neurons in the hidden layer and the learning rate are determined through grid search, while the transfer functions of the hidden layer and the output layer are determined based on empirical analysis of the target error iteration limit. The topological structure of the RBF neural network is shown in Figure 10. The final selection of the hyperparameters for the RBF neural network is presented in Table 11.

#### 4.3.2. BP Neural Network

Back propagation neural network (BPNN) is a typical feedforward multi-layer perceptron (MLP) that optimizes weights and biases through the backpropagation algorithm; it was first proposed by Rum et al. [69] in 1986. The network training is combined with the Levenberg–Marquardt (LM, trainlm) method. This method achieves rapid second-order optimization of the sum of squared errors, with fast convergence speed and high accuracy.

In this study, BP is composed of an input layer, a hidden layer, and an output layer, with the number of hidden layers ranging from one to multiple layers. The input layer receives the output from the RBF layer. Hidden layer, whose number of nodes is a hyperparameter (search range for the grid [10, 15, 20, 25, 30]). Output layer, which has two nodes that correspond to the two indicators for predicting the concrete’s impact resistance performance.

The activation function of the hidden layer is the tansig function, with an output range of (−1, 1). The transfer function of the output layer is purelin, which directly outputs continuous numerical values. The transformation of the hidden layer is nonlinear, while the transformation of the output layer is linear. The BP neural network algorithm is shown in Equations (10)–(12).

(10) $$Z_{1}=XW_{1}^{⊤}+1_{n}b_{1}^{⊤},\;A_{1}=f\left(Z_{1}\right)$$

(11) $$\hat{Y}=A_{1}W_{2}^{⊤}+1_{n}b_{2}^{⊤}$$

(12) $$L=\frac{1}{n}∥\hat{Y}-Y{∥}_{F}^{2}$$

where $X\&\in R^{n\times d}$ is the input matrix, $W_{1}\&\in R^{N_{h}\times d}$ is the hidden layer weights, $b_{1}\&\in R^{N_{h}\times 1}$ is the hidden layer bias, f(⋅) is the activation function, $A_{1}\&\in R^{n\times N_{h}}$ is the hidden layer output.

**Figure 11:**
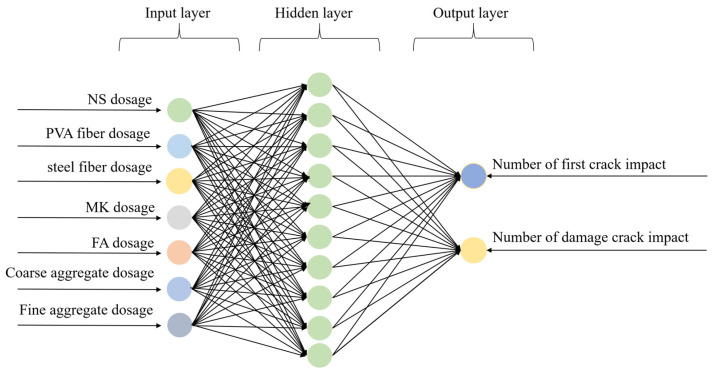
Structure of BP neural network.

The selected hyperparameters for this study are shown in Table 12, and the structure of the BP neural network is depicted in Figure 11.

#### 4.3.3. RBF-BP Neural Network

Since RBF only trains the weights of the output layer, its training speed is fast. The structure is simple, and it is suitable for small sample problems. The local response characteristic enables it to have strong mapping ability and can better fit local nonlinear relationships. However, it is sensitive to high-dimensional input. The advantage of the BP neural networks is that they can approximate any nonlinear function. The disadvantage of BP neural networks is that the training speed is slow, they are prone to getting stuck in local optimal solutions, and their generalization ability significantly deteriorates when there is noise.

Given the above circumstances, this study combines the RBF-BP two networks to make up for the shortcomings of the two algorithms. In the RBF-BP, the RBF layer is placed before the BP layer [70]. By offering nonlinear mapping capabilities, the BP layer improves global fitting accuracy. It further resolves an issue with the standalone RBF network. BP output layer relies on linear mapping, which hinders capturing overall complex nonlinearities. The RBF layer first performs local feature mapping, which reduces the training difficulty of the BP layer. At the same time, it improves the convergence speed, thereby solving the problem that the single BP is prone to getting stuck in a local optimal solution, has a slow training process, and is sensitive to the initial weights, resulting in unstable convergence. The RBF layer was placed before the BP layer based on principles of function approximation and feature representation. An RBF network performs a nonlinear transformation of the input space through radial basis functions, which can be interpreted as a basis expansion that maps complex, highly nonlinear relationships into a higher-dimensional feature space with improved separability. This transformation allows local input patterns to be captured effectively by Gaussian kernels, resulting in structured and smoother feature representations. Placing the BP network after the RBF layer enables the BP component to learn the global mapping between these extracted features and the output through gradient-based optimization, rather than directly operating on the raw input space. Such a sequence improves the conditioning of the learning problem, particularly under small-sample conditions, and allows the hybrid model to combine localized nonlinear representation with flexible parametric learning, leading to more stable and reliable predictions of NSHFGPC performance.

**Figure 12:**
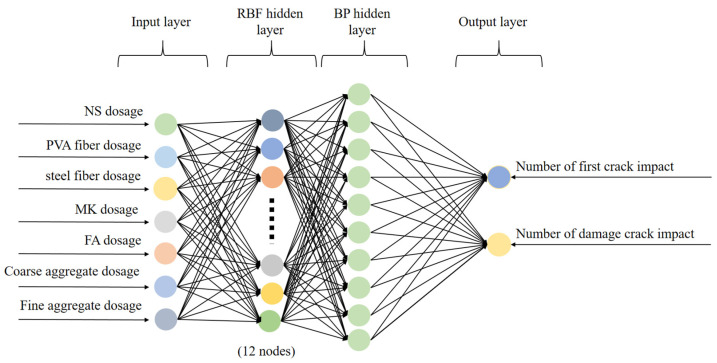
Structure of the RBF-BP neural network.

The selection of hyperparameters is summarized in Table 13, whereas the topological structure of the RBF-BP neural network is illustrated in Figure 12.

### 4.4. Model Training

Before constructing the neural network algorithm, in an effort to mitigate the effects of feature scale discrepancies on neural network training processes [71], the input and output data were processed using Min–Max normalization, mapping them linearly to the [0, 1] interval. Min–Max normalization was adopted instead of Z-score standardization to ensure compatibility with the activation functions used in the neural network architectures. In the BP and RBF–BP models, the hidden layer employs the Tansig activation function, whose effective operating region is bounded. Scaling input variables to the [0, 1] range helps keep neuron activations within sensitive regions of the Tansig function, thereby reducing saturation effects and improving gradient propagation. In addition, the Gaussian radial basis functions used in the RBF layers are distance-sensitive, making feature scaling essential to prevent variables with large magnitudes from dominating the training process. Although Min–Max normalization can be sensitive to outliers, basic exploratory data analysis based on descriptive statistics and range inspection indicated that no extreme outliers were present in the dataset. Under these conditions, Min–Max normalization provides a stable and effective preprocessing strategy for the adopted neural network structures. After the prediction is completed, to facilitate the interpretation and comparison of the results, the normalized predicted values are inversely normalized (re-normalized) back to the original scale. Normalization and de-normalization can be expressed, respectively, by Equations (13) and (14).

(13) $$\begin{array}{c} x_{n}^{\left(j\right)}=\frac{x^{\left(j\right)}-x_{min}^{\left(j\right)}}{x_{max}^{\left(j\right)}-x_{min}^{\left(j\right)}} \end{array}$$

(14) $$\begin{array}{c} {\hat{y}}^{\left(j\right)}=y_{n}^{\left(j\right)}\cdot \left(y_{max}^{\left(j\right)}-y_{min}^{\left(j\right)}\right)+y_{min}^{\left(j\right)} \end{array}$$

where $x^{\left(j\right)}$ is the value of the *j*-th original feature, $x_{min}^{\left(j\right)}$ and $x_{max}^{\left(j\right)}$ are the minimum and maximum values of the *j*-th feature in the training set, $x_{n}^{\left(j\right)}$ is the value after normalization to the range of [0, 1], $y_{n}^{\left(j\right)}$ is the normalized output value predicted by the model, $y_{max}^{\left(j\right)}$ and $y_{min}^{\left(j\right)}$ are the maximum and minimum values of the *j*-th output variable in the training set, and ${\hat{y}}^{\left(j\right)}$ is the predicted value restored to its original units.

For the purpose of ensuring the generalization ability of the RBF-BP neural network, this study adopts the grid search combined with K-fold cross-validation to automatically select the hyperparameters. Within the preset range of hyperparameter candidates, the *R*^2^ (coefficient of determination) of each parameter group on the validation set is calculated through K-fold cross-validation. The 13 samples represent a relatively small sample size for the training of the neural network. In this small-data scenario, K-fold cross-validation was adopted to reduce overfitting and improve the robustness of model evaluation. By repeatedly training the network on different subsets of the data (each fold using a reduced portion of the samples for validation), the model performance was assessed across multiple train–validation splits rather than relying on a single partition. This process limits the tendency of the network to fit noise specific to a particular subset and allows hyperparameters to be selected based on averaged performance across all folds. Finally, the parameter group with the best performance is selected as the model’s hyperparameters. The advantage of this method lies in, on the one hand, its avoidance of the randomness caused by the random division in single training/validation, making the results more stable; on the other hand, it systematically explores the parameter space, improving the model’s generalization ability under different data distributions.

**Figure 13:**
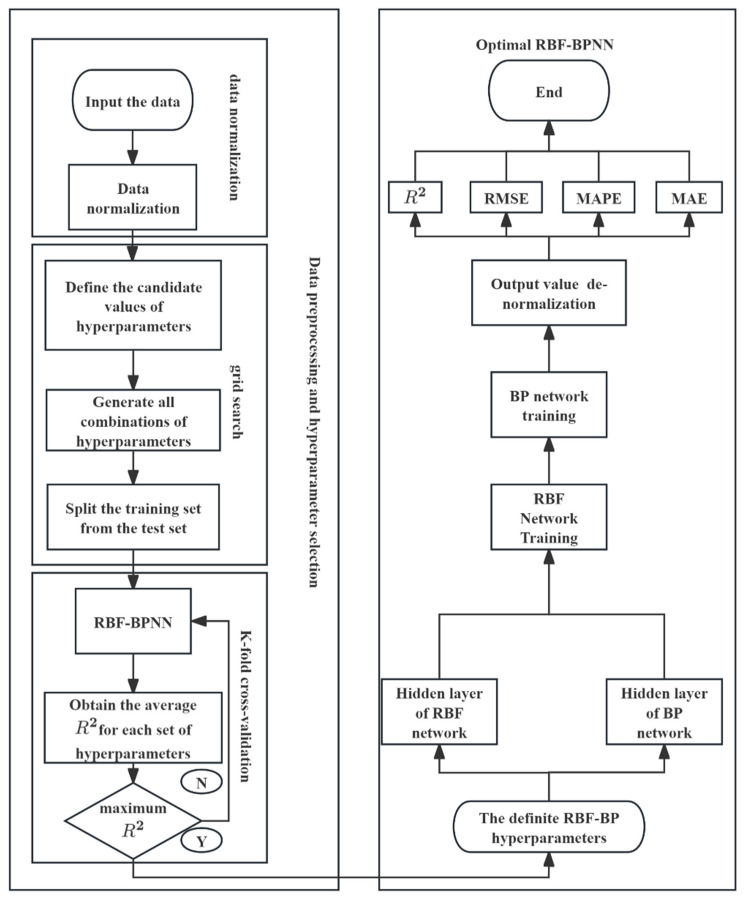
Structure flowchart of the RBF-BP neural network algorithm.

The following is a detailed explanation of the training process of the RBF-BP neural network. First, the seven input data points are normalized to the range of [0, 1] to reduce the errors caused by the differences in input data scales and correlations and also to decrease the possibility of model overfitting. Then, a grid search was conducted on the three hyperparameters of RBF and BP hidden layer neurons and learning rate. The maximum number of iterations and the target error were determined based on experience. K-fold cross-validation was performed for all combinations of hyperparameters. During the cross-validation, the training set and test set were divided. First, the RBF layer was trained, mapping the input into high-dimensional features. Then, the BP layer was used to fit the RBF output to the target output. All hyperparameter combinations were calculated in parallel using the parfor function, and the *R*^2^ was recorded as the primary evaluation metric [72]. Following the standard protocol for model selection, the parameter combination yielding the maximum *R*^2^ was identified as the optimal configuration [73]. Subsequently, the final RBF-BP network was trained using these optimized hyperparameters. Figure 13 illustrates the structure flowchart of the RBF-BP neural network algorithm.

### 4.5. Model Comparison Between RBF-BP and Simple Models

In order to verify the advantages of the RBF-BP composite neural network proposed in this study, the RBF-BP prediction performance was compared with that of simplified statistical approaches such as MLR and SVR.

**Figure 14:**
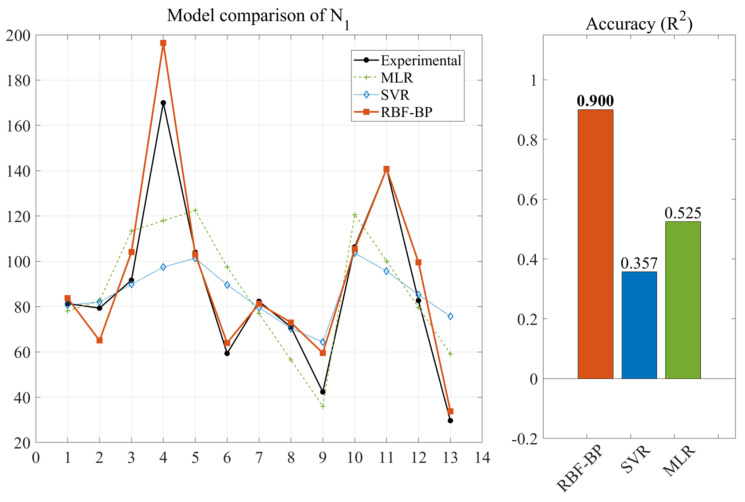
Model comparison of *N*_1_.

**Figure 15:**
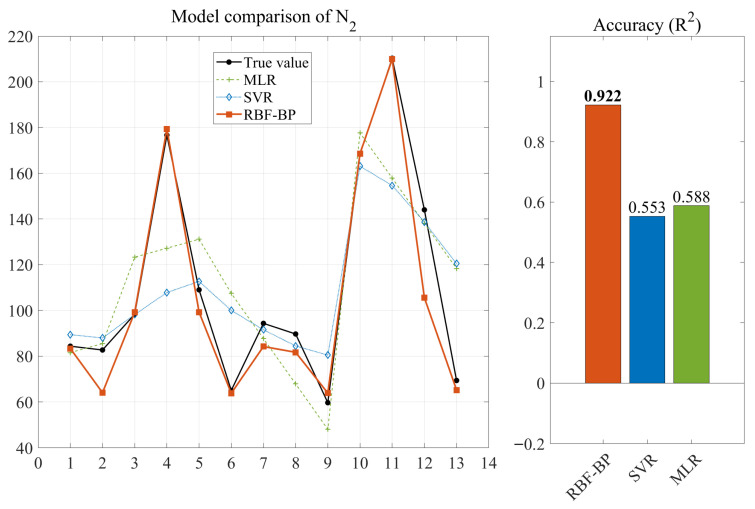
Model comparison of *N*_2_.

The MLR model was implemented using the fitlm function within the Statistics and Machine Learning Toolbox. To ensure a fair comparison, the same normalized dataset (N = 13) used for the RBF-BP network was input into the MLR framework. The model estimated the partial regression coefficients for the seven design parameters using the Ordinary Least Squares (OLS) method. During execution, the program monitored for rank deficiency, a common indicator of multicollinearity in high-dimensional concrete mix designs, to evaluate the validity of linear mapping. The SVR model was constructed via the fitrsvm function. To capture the nonlinear impact resistance, a Gaussian Radial Basis Function kernel was employed to map the input features into a high-dimensional space. The program optimized the model hyperparameters to establish an optimal insensitive tube that balances complexity and prediction error. To maintain consistency, the SVR output was re-mapped to the original scale using the mapminmax inverse function, allowing for a direct comparison of performance metrics with the hybrid model. Figure 14 and Figure 15 present the comparative results for the output parameters *N*_1_ and *N*_2_, respectively. Each figure includes the predicted values and the corresponding *R*^2^ metrics for the MLR, SVR, and RBF-BP models.

As illustrated in Figure 14 and Figure 15, the RBF-BP network demonstrates the superior fitting performance, with *R*^2^ values for *N*_1_ and *N*_2_ reaching 0.900 and 0.922, respectively. Furthermore, the prediction accuracy of the MLR model outperforms that of the SVR model. Several factors contribute to the inferior performance of MLR and SVR compared to the RBF-BP network. As a fundamental statistical approach, MLR assumes a linear superposition relationship among variables. However, the micro-filling effect of NS and the toughening mechanism of FA involve highly intricate interactions within the geopolymer matrix, which mathematically manifest as strong nonlinearity. Furthermore, in an environment with multiple input dimensions and limited samples, multicollinearity among independent variables easily leads to rank deficiency during the solution process. The above two points render the regression coefficients unstable and lack generalization ability. SVR employs kernel functions for non-linear mapping. Data sparsity hinders the formation of a robust insensitivity region. The model ignores local fluctuations to ensure smoothness. This limitation causes severe underfitting on discrete experimental data. The hybrid neural network achieves superior accuracy. The radial basis layer extracts local features. The back-propagation layer optimizes global weights. This dual strategy overcomes data limitations. Therefore, choosing the RBF-BP composite neural network as the prediction method in this paper is appropriate.

## Figures and Tables

**Table 1:** Correspondence between the specimens and the mix number.

Mix no.	**1**	**2**	**3**	**4**	**5**	**6**	**7**
Specimen	C	N-0.5	N-1.0	N-1.5	N-2.0	NP-0.2	NP-0.4
Mix no.	**8**	**9**	**10**	**11**	**12**	**13**	
Specimen	NP-0.6	NP-0.8	NSP-0.2	NSP-0.4	NSP-0.6	NSP-0.8	

**Table 2:** Comparison of prediction accuracy of three neural networks.

Neural Network Models	Impact Resistance	*R* ^2^	*RMSE*	*MAPE*	*MAE*
RBF-BP neural network	*N*_1_	0.9003	11.3897	10.6732	7.9545
	*N*_2_	0.9222	12.8130	7.4373	7.6736
RBF neural network	*N*_1_	0.7491	18.0682	14.0493	12.1882
	*N*_2_	0.8294	18.9811	10.4007	12.6423
BP neural network	*N*_1_	0.5955	22.9440	29.0800	17.8986
	*N*_2_	0.5883	29.4846	23.8382	24.0762

**Table 3:** Chemical compositions of MK and FA.

Composition (wt.%)	Al_2_O_3_	SiO_2_	Fe_2_O_3_	Na_2_O + K_2_O	CaO + MgO	SO_3_	Other
FA	24.47	60.98	6.70	–	5.58	0.52	1.75
MK	43.0	54.0	≤1.3	≤0.7	≤0.8	–	≥0.2

**Table 4:** Physical properties of FA.

Density (g/cm^3^)	Bulk Density (g/cm^3^)	Standard Consistency (%)	Water Demand Ratio (%)
2.16	0.77	47.1	105

**Table 5:** Physical properties of MK.

Availability of Lime (mL)	Average Particle Size (µm)	Activity Index (%)	Whiteness (%)	Ignition Loss (%)
1350	1.2	12	75	0.5

**Table 6:** Physical properties of NS.

Content of SiO_2_ (%)	Bulk Density (g/cm^3^)	Specific Surface Area (m^2^/g)	Average Particle Size (nm)	pH
99.5	0.035	200	30	6

**Table 7:** Physical properties of PVA.

Specific Gravity (g/cm^3^)	Standard Length (mm)	Strength of Extension (MPa)	Eongation at Break (%)
1.32	9	1400	15

**Table 8:** Mix proportions of NSHFGPC for train set [63].

Specimen	MK	FA	Water Glass	NaOH	Water	Coarse Aggregate	Fine Aggregate	NS	PVA Fiber	Steel Fiber
	kg/m^3^	kg/m^3^	kg/m^3^	kg/m^3^	kg/m^3^	kg/m^3^	kg/m^3^	%	%	%
C	273	195	286	53.2	79	1072	577	0	0	0
N-0.5	272	194	286	53.2	79	1072	577	0.5	0	0
N-1.0	270	193	286	53.2	79	1072	577	1.0	0	0
N-1.5	269	192	286	53.2	79	1072	577	1.5	0	0
N-2.0	268	191	286	53.2	79	1072	577	2.0	0	0
NP-0.2	269	192	286	53.2	79	1072	577	1.5	0.2	0
NP-0.4	269	192	286	53.2	79	1072	577	1.5	0.4	0
NP-0.6	269	192	286	53.2	79	1072	577	1.5	0.6	0
NP-0.8	269	192	286	53.2	79	1072	577	1.5	0.8	0
NSP-0.2	269	192	286	53.2	79	1021	549	1.5	0.2	1.0
NSP-0.4	269	192	286	53.2	79	1021	549	1.5	0.4	1.0
NSP-0.6	269	192	286	53.2	79	1021	549	1.5	0.6	1.0
NSP-0.8	269	192	286	53.2	79	1021	549	1.5	0.8	1.0

**Table 9:** Values of *N*_1_, *N*_2_, and *N* [63].

Mix No.	*N*_1_	*N*_2_	*N*
C	81.33	84.33	3.00
N-0.5	79.33	82.67	3.33
N-1.0	91.67	98.33	6.66
N-1.5	170.00	176.67	6.67
N-2.0	104.00	109.00	5.00
NP-0.2	59.33	65.00	5.67
NP-0.4	82.33	94.33	12.00
NP-0.6	71.00	89.67	18.67
NP-0.8	42.33	59.67	17.33
NSP-0.2	106.33	168.33	62.00
NSP-0.4	141.00	210.33	69.33
NSP-0.6	82.67	144.00	61.33
NSP-0.8	29.67	69.33	39.67

**Table 10:** Components of grid search parameters.

Components of Grid Search Parameters					
Number of neurons in the RBF hidden layer	6	8	10	12	14
Number of neurons in the BP hidden layer	10	15	20	25	30
Learning rate	0.05	0.1	0.3	0.5	0.7

**Table 11:** Parameters for the RBF neural network.

Parameter Types	Neurons of Hidden Layer	Target Error	Iteration Limit	Learning Rate	Transfer Function of Hidden Layer	Transfer Function of Hidden Layer
value	12	10^−7^	15,000	0.05	Gauss	Linear

**Table 12:** Parameters for the BP neural network.

Parameter Types	Hidden Layer Neurons	Target Error	Iteration Limit	Activation Function of Hidden Layer	Transfer Function of Output Layer
value	10	10^−7^	15,000	Tansig	Puerlin

**Table 13:** Parameters for RBF-BP neural network.

Parameters Types	Hidden Layer Neurons of RBF	Hidden Layer Neurons of BP	Hidden Layer Excitation Function of RBF	Hidden Layer Excitation Function of BP	Transfer Function of Output Layer
Value	12	10	Gauss function	Tansig	Purelin

## Data Availability

No new data were created or analyzed in this study.
